# Louisville Medical College

**Published:** 1850-10

**Authors:** 


*Louisville Medical College.*—Daniel Drake, M. D., has been elected to the chair of medicine in this institution, made vacant by the resignation of Prof. Bratlett. The chair of surgery is not yet filled.

